# Five‐year follow‐up report: Box lesion radiofrequency ablation procedure for atrial fibrillation under video‐assisted thoracoscope

**DOI:** 10.1002/ccr3.4837

**Published:** 2021-12-05

**Authors:** Yupeng Ji, Li He, Zeyi Cheng, Jun Shi, Lulu Liu, Yingqiang Guo

**Affiliations:** ^1^ Department of Cardiovascular Surgery Sichuan University West China Hospital Chengdu China

**Keywords:** atrial fibrillation, epicardial ablation, minimally invasive surgery

## Abstract

We report the initial 5‐year follow‐up of a novel mini‐invasive procedure for epicardial ablation for the treatment of atrial fibrillation. The initial 5‐year survival rate is acceptable and comparable with that of hybrid ablation. And this shared procedure has the advantages of shorter operation time and less surgical trauma.

## Graphical abstract

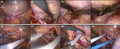

## 1 INTRODUCTION

Radiofrequency ablation procedure has been applied to the treatment of atrial fibrillation (AF), while limited articles referred to its long‐time efficacy. The objective of this study was to report the initial 5‐year follow‐up of a novel mini‐invasive procedure for epicardial ablation applied in our center. Thirty‐one patients with symptomatic atrial fibrillation were consecutively enrolled in our study, which had unsuccessful drug therapy or endocardial ablation or were intolerant to antiarrhythmic drugs. The surgery performance included three 5–12‐mm holes on each side of the chest wall. A bipolar radiofrequency device guided by navigators was applied for the electrical isolation of pulmonary veins and the posterior wall of left atrium. And a surgical stapler was used to excise the left atrial appendage. The procedure was successfully performed on every patient, and its median time was 115 min. No death, conversion to sternotomy or thoracotomy, phrenic paralysis, stroke, pneumonia, transfusion, or pacemaker insertion occurred. Postoperatively, five patients underwent electrical cardioversion and returned to sinus rhythm remaining beyond the discharge. Twenty‐nine enrolled patients fulfilled the 5‐year follow‐up, and the arrhythmia‐free survival rates after one epicardial procedure were 62.9%, 55.9%, 52.4%, and 45.4% at 1, 2, 3, and 5 years respectively. Despite the sharp decrease of twenty‐one percent in the second 6 months, the initial 5‐year survival rate of the applied procedure is acceptable and comparable with that of hybrid ablation. And this shared procedure is one of the reported procedures least time‐consumptive and harmful.

Atrial fibrillation (AF) is the most common arrhythmia and is complicated with thrombosis, atrial pumping dysfunction, and stroke. As the pharmacological treatment of AF is not always effective but sometimes intolerable, the surgical management comes necessarily. The Cox maze III procedure was described by Cox et al^1^, ^2^, ^3^ developed with new ablation sources realizing the commendable efficacy of surgical intervention. Despite incorporating this golden procedure for concomitant AF with valvular disease, the pursuit of less invasive techniques treating AF, especially the lone AF, never fade. As mechanism research highlights the stimuli from pulmonary veins (PVs) and posterior wall of left atrium, catheter ablation is first introduced by cardiologist and subsequently amended as PVs circular isolation and extra linear lesion electrically blocking the posterior wall.^4^, ^5^, ^6^, ^7^ However, the general results of catheter‐based ablation techniques used for lone AF are relatively disappointing, as the arrhythmia‐free survival rate at 5 years is less than 30%.^8^, ^9^

Epicardial ablation also named video‐assisted thoracoscopic surgery (VATS) was reported in 2005, and this minimally invasive procedure allows AF management through surgical approach on beating hearts.^10^ Encouraged by this technique, cardiac surgeons are more willing to deal with AF. According to the Society of Thoracic Surgeons Adult Cardiac Surgery Database, from 2005 to 2010 stand‐alone surgical ablations increased significantly from 552 to 1041 cases, and the off‐CPB procedure as the majority has better performance in avoiding stroke, renal failure, reoperation for bleeding, and other complications.^11^ With the evolvement of techniques, there are other two minimally invasive ablation procedures for AF recently introduced as hybrid ablation by Pison et al in 2012 and minimally invasive Cox maze procedure by Ad et al in 2013 respectively.^12^, ^13^ In this study, we implicate one of the epicardial procedures as the least invasive and time‐saving method to share our single‐center experience.

## 2 MATERIALS AND METHODS

### 2.1 Patient selection

Thirty‐one patients with symptomatic atrial fibrillation were consecutively enrolled in our study. The inclusion criteria were along with our previously published article as (1) recurrent AF and (2) AF refractory to antiarrhythmic drugs, while the excluded criteria were: (1) previous pulmonary and cardiac surgery, (2) underlying cardiovascular or pulmonary diseases other than AF, (3) atrial thrombi or persistent left superior vena cava, for which preoperative transthoracic echocardiography and computed tomography (CT) scan were made. Actually, every enrolled patient was also allocated to lung function test and coronary angiographic analysis checking whether there was any need for extra deposition. And following the Heart Rhythm Society, European Heart Rhythm Association, and European Cardiac Arrhythmia Society consensus statement they were all classified into paroxysmal, persistent, and longstanding persistent AF.^14^ Our study was approved by the Clinical Research and Biomedical Ethical Committee of West China Hospital Sichuan University.

### 2.2 Surgical procedure

**FIGURE 1:**
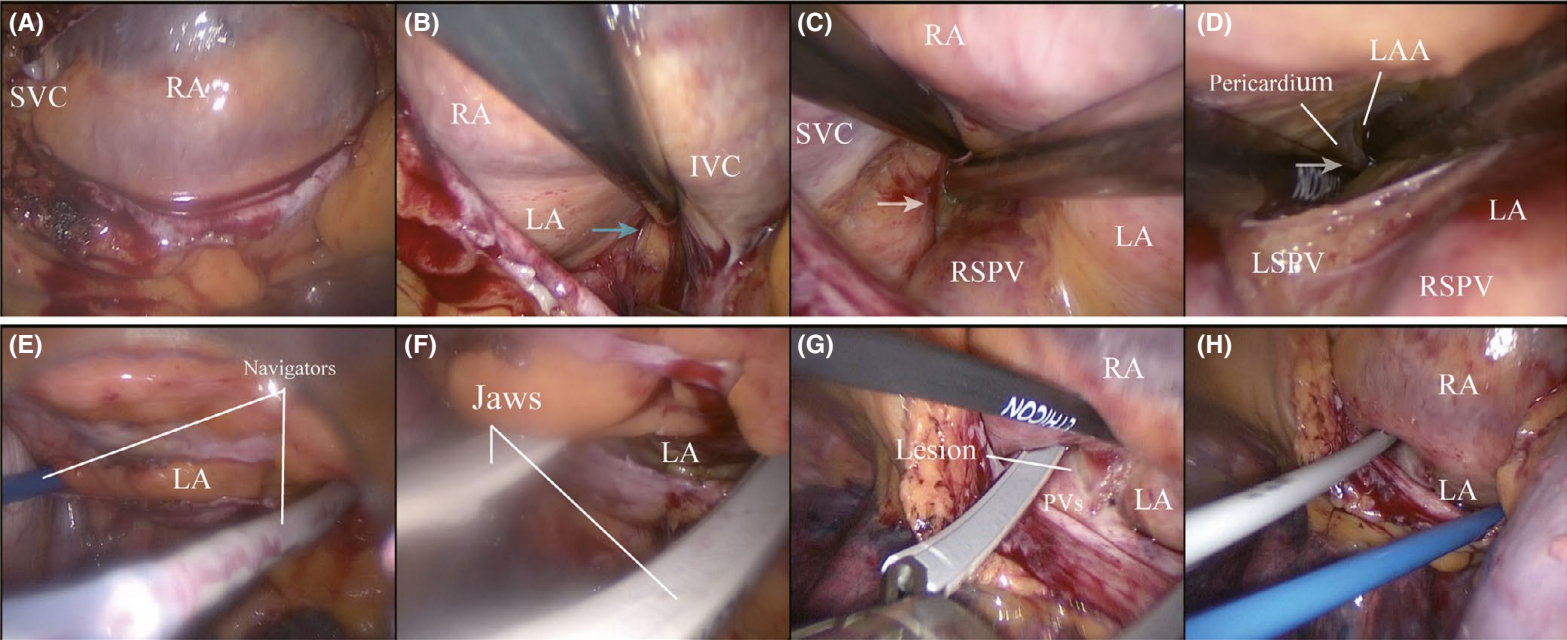
Continuous intraoperative endoscopic view of the ablation procedure. (A) Open the right‐sided pericardial sac. (B) Blunt dissection of the oblique sinus (blue arrow). (C, D) Blunt dissection of the transverse sinus (white arrow). (E) Unveiling the tips of two navigators on the left side. (F) Left side ablation. (G, H) Right side ablation. IVC‐Inferior vena cava; LA, Left atrium; LAA, Left atrial appendage; LSPV, Left superior pulmonary vein; PV, Pulmonary vein; RA, Right atrium; RSPV, Right superior pulmonary vein; SVC, Superior vena cava

**FIGURE 2:**
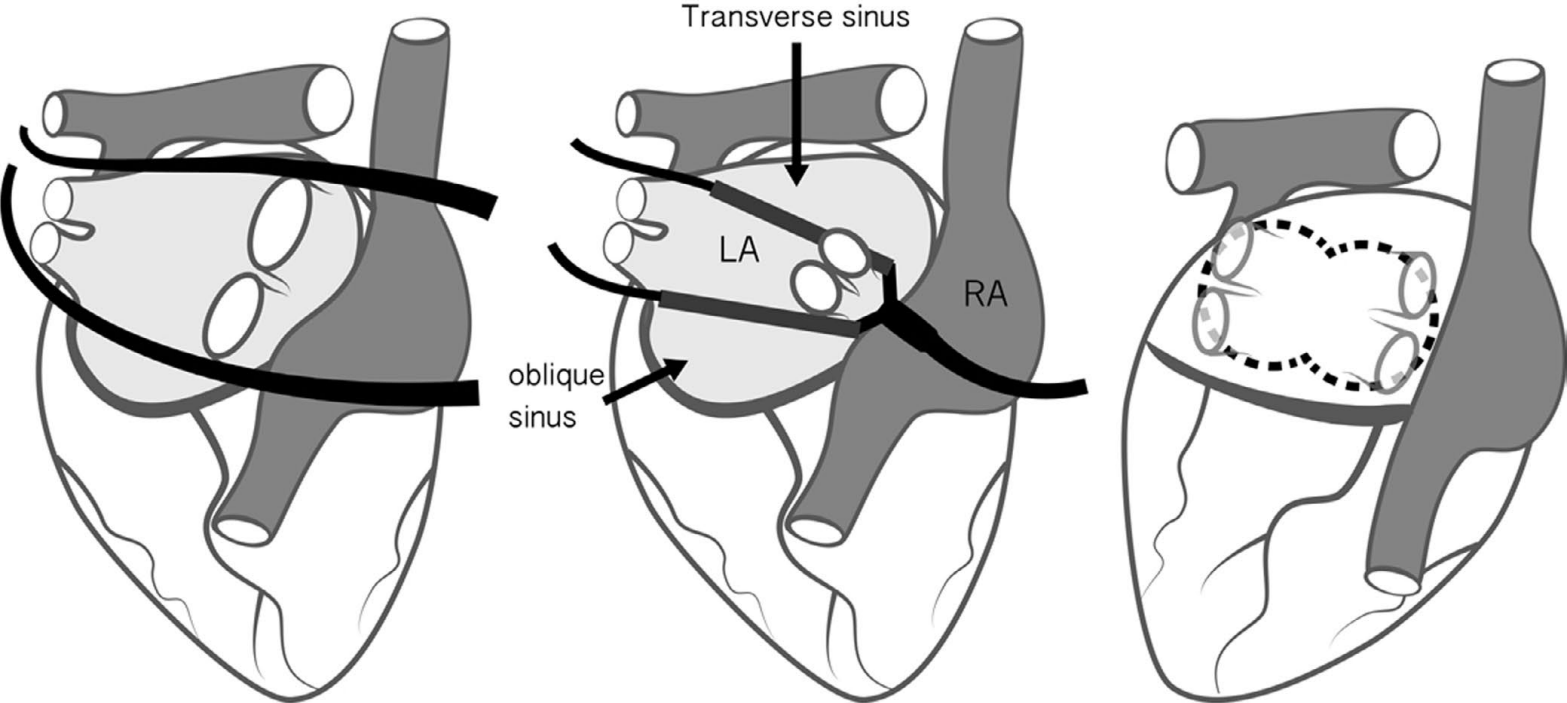
Schematic drawing of the posterior aspect of the heart. Two navigators were positioned in the transverse and oblique sinuses to guide the ablation device. The dashed line in the figure shows the box lesion created after ablation on both sides. LA, Left atrium; RA, Right atrium

The procedure has been described earlier and will briefly be reviewed here.^15^ The patients assumed the supine position with both upper arms dorsiflexed 15° to make bilateral chest walls be exposed completely and were intubated with double‐lumen endotracheal tubes under the induction of general anesthesia. As a start, since the right lung deflated, a 5‐mm trocar was introduced into the fourth intercostal space in the anterior axillary line for CO2 insufflation at approximately 8 to 12 mmHg. And we also constructed other two 5‐mm ports respectively in the third and fifth intercostal space at the anterior axillary line. Subsequently, the 12‐mm port as working port was transformed from the first 5‐mm port. To open the right‐sided pericardial sac, a lesion paralleling to the phrenic nerve was made from the superior vena cava (SVC) to the inferior vena cava (IVC) about 2‐cm anterior. When pericardium was open, the transverse and oblique sinuses were exposed by blunt dissection, through which each Cardioblate Navigator (Medtronic) could be put respectively. In the next, the ventilation of the right lung was restored, while its left counterpart was deflated. Through the left corresponding ports, the left‐sided pericardium was opened to unveil the tips of two navigators, which then were attached to jaws of the Cardioblate Gemini‐s bipolar radiofrequency isolator (Medtronic). The radiofrequency ablation started from the left side and moved forward to the right side, fulfilling the continuous box lesion which isolated the pulmonary veins and posterior left atrial wall completely. At last, a single 28 French Chest Tube was placed in each pleural space through the most inferior ports, and the patient was transferred to the intensive care unit for recovery (Figures 1 and 2).

### 2.3 Rhythm control and anticoagulation management

Continuous cardiac rhythm monitoring was sustained for another 48 h and then replaced by daily 12‐lead electrocardiograms. While for patients with recurrent AF and postoperative Af (atrial flutter), electrical cardioversion was applied. Amiodarone the suggested Antiarrhythmic drug (AAD) was generally implicated postoperatively and recommended as discontinued 3 months after the operation only if the sinus rhythm remained. Similarly, the warfarin could be stopped after 3 months as if the thromboembolic risk is low, which was started from postoperative day 2 targeting the INR range from 1.5 to 2.5 (an experience of our center).

### 2.4 Follow‐up

All patients were followed up at postoperation 1 month, 3 months, 6 months, 1 year, 3 years, and 5 years and for each time 24 h Holter monitoring was performed, so as to patients with normal ECG but AF‐relating symptoms. Long‐term success was defined as freedom from AF/Af/AT (atrial tachycardia) recurrences following the 3‐month blanking period through a minimum of 36‐month from the date of the ablation procedure in the absence of Class I and III antiarrhythmic drug therapy according to 2017 HRS/EHRA/ECAS/APHRS/SOLAECE expert consensus statement on catheter and surgical ablation of atrial fibrillation. And among the enrolled patients, twenty‐nine patients fulfilled the video‐assisted thoracoscopic ablation and the follow‐up of 5 years.

### 2.5 Statistical analysis

Normal values were expressed as mean ± standard deviation (SD), non‐normal values as median and IQR, and categorical variables as percentages. The Mantel‐Haenszel chi‐square was employed to establish differences among groupings. Statistical analysis was performed using SPSS release 22.0. *p*‐values less than 0.05 were considered significant.

## 3 RESULTS

### 3.1 Baseline characteristics

Baseline characteristics were presented in Table 1. Among the thirty‐one enrolled patients, twenty patients had paroxysmal AF, eight patients had persistent AF, and the left three had longstanding persistent AF. The median follow‐up duration was 60 months (range, 2 to 384 months). And the mean left atrial diameter of patients with paroxysmal AF was 39.06 ± 4.45, and there was no significant difference when compared with 41.6 ± 3.80 of patients with nonparoxysmal AF (*p* = 0.14). AADs were not that effective or intolerable to all patients. And previous transcatheter endocardial ablation was documented with five patients with recurrent AF.

**TABLE 1:** Baseline characteristics (*n* = 31) CHADS_2_, congestive heart failure, hypertension, age, diabetes, and prior stroke or transient ischemic attack score

Characteristic	Mean ± SD or *n*
Age, y	66.17 ± 8.32
Gender (male/female)	23/8
Hypertension	12
Diabetes	2
Cardiac disease	
Patent foramen ovale	2
Patent ductus arteriosus	1
Coronary heart disease	1
Valvular diseases	4
Congestive heart failure	4
Prior TIA/stroke	5
AF duration [months]	60 [2–384]
Type of AF	
Paroxysmal AF	20
Persistent AF	8
Longstanding Persistent AF	3
CHADS_2_ score	2 [0–6]
Prior catheter ablation	5
Antiarrhythmic drugs	
β‐blocker	18
Cedilanid	2
Amiodarone	8
Propafenone	1
Anticoagulation drugs	
Aspirin	6
Clopidogrel	4
Warfarin	2
LAD	40 ± 4.28
EF	63 ± 10.27

Abbreviations: EF, ejection fraction; LAD, left atrial diameter; TIA, transient ischemic attack.

### 3.2 Perioperative data

The epicardial ablation was successfully performed in every patient. And the median procedure time was 115 min. The procedure time would decrease as the more familiar the performing surgeon getting, and the shortest documented time was 45 min obtained in the later practice. No death and conversion to sternotomy or thoracotomy occurred intraoperative. And no phrenic paralysis, stroke, pneumonia, transfusion, or pacemaker insertion was documented either. Electrical conversion was performed as patients with recurrent AF or emerging AFL after the operation during the hospital stay turning out to be effective. The overall hospital stay time ranged from 5 to 12 days with the median as 8 (Table 2).

**TABLE 2:** Perioperative data (*n* = 31)

Perioperative data	Median (range) or *n* (%)
Procedure time	115 (45–180)
Extubation time	5 (3–18)
ICU‐stay time	1 (1–2)
Hospital‐stay time	8 (5–12)
Electrical conversion	5 (16.13)
Postoperative sinus rhythm	26 (83.87%)

### 3.3 Arrhythmia‐free survival after one procedure

**FIGURE 3:**
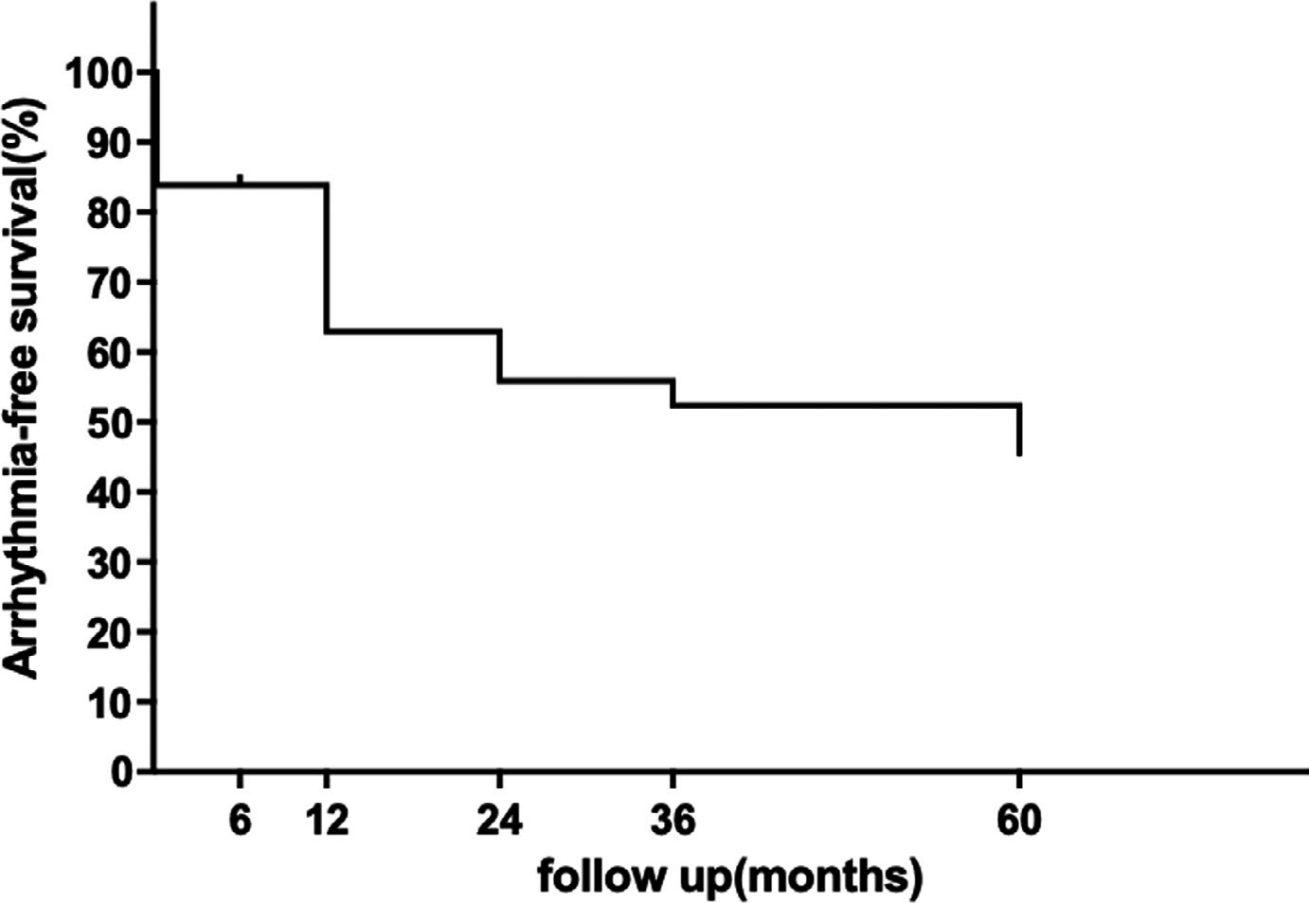
Kaplan‐Meier curve represents the percentage of patients free from AF, atrial flutter, and atrial tachycardia without AADs up to 5 years after surgery. The sharpest drop of the curve lies between month 6 and month 12

**FIGURE 4:**
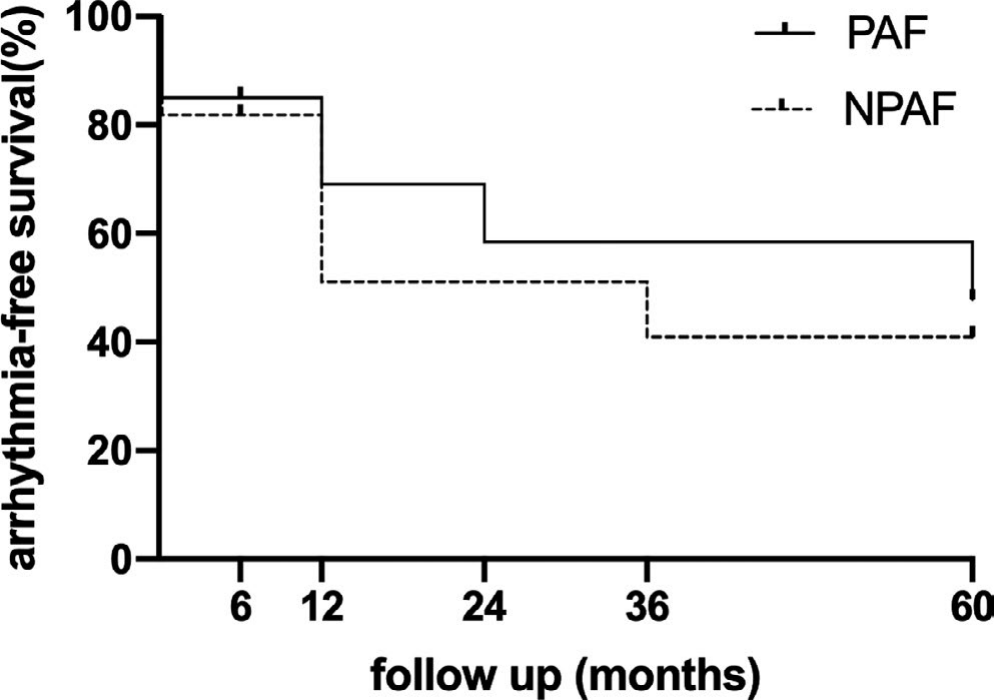
Single‐procedure survival rates of patients with PAF (paroxysmal atrial fibrillation) and NPAF (nonparoxysmal atrial fibrillation) are delineated. And there is no significant difference between the two curves (*p* = 0.66)

During the postoperative hospital stay, five patients underwent electrical cardioversion and returned to sinus rhythm (SR), which remained until discharge. As the follow‐up settled above and the loss of two patients both at month six, the arrhythmia‐free survival rates after one epicardial procedure were 62.9%, 55.9%, 52.4%, and 45.4% at 1, 2, 3, and 5 years respectively (Figure 3). As early studies described the distinct efficiencies of epicardial ablation for different patients, the survival rates of paroxysmal AF and nonparoxysmal AF patients were shown in Figure 4. With these data, we found that the peak recurrence time of this procedure occurred in the second 6 months after operation, during which the overall survival rate decreased by almost 21%.

## 4 DISCUSSION

Given the previously published data, the annual count of stand‐alone surgical procedures performed in STS‐documented medical centers is doubled in 2010 when compared with that of 5 years ago.^11^ And since first reported in 2005, epicardial ablation has been widely accepted and conducted for its evident safety and promising efficacy.^10^ In our research, with the implication of Cardioblate Navigators (Medtronic) and Cardioblate Gemini‐s bipolar radiofrequency isolator (Medtronic), we are able to apply smaller ports and further minimalize the invasiveness of the operation.

During the 15 years practicing of epicardial ablation, many remarkable improvements are made, one of which worth being highlighted is the evolving of the lesion set. Early research based on catheter ablation indicates that besides PVI, extra linear lesions in the posterior wall of left atrium are essential for the maintenance of sinus rhythm after endocardial ablation in patients with permanent AF, left atrial dilatation, and valvular heart disease.^5^, ^7^ And in early studies about epicardial ablation, as technology limits the primary lesion set including separated circulating isolation of the left and right PVs have avoided these essential linear lesions intentionally or unintentionally.^10^, ^16^, ^17^ Then followed with subsequent addition of roofline and dispensable inferior line, while there are three main obstacles remained when completing the mitral annulus connecting lesion through thoracoscopic approach at the sites corresponding to Cox maze III set: (1) the nonvisibility of posterior wall of left atrium in the hollow space behind it; (2) the inevitable risk damaging the circumflex coronary artery; (3) and the possible result of incomplete lesion as the coronary sinus the dissatisfactory epicardial mark may be up to 13 mm away from the mitral annulus. Edgerton et al^18^. reorients the mitral annulus connecting lesion to the doom of the left atrium from the left fibrous trigone at the anterior mitral valve annulus to the roof line together with the mentioned epicardial lesions described as the Dallas lesion set. In our study as formerly described, the symmetric lesions performed oppositely but combined forming the continuous isolation of the PVs and main portion of posterior left atrium wall, and in our later practice, the additional mitral annulus connecting line has been performed.

Many researchers reproach the recovered pulmonary vein conduction accounting for the recurrence of ATa (Atrial tachyarrhythmia) after PVs isolation, which is caused by incomplete and non‐transmural ablation lines.^19^, ^20^, ^21^, ^22^ They also advocate the importance of electrophysiology confirmation after the ablation procedure ensuring the endpoint as bidirectional conduction block and noninducibility of atrial fibrillation, which leads to the promising ATa episode free rate without AAD reaching ninety percent for the mean follow‐up duration of 6 months.^19^ Although endocardial mapping has been widely accepted by catheter ablation, it is impossible to realize under the non‐heparinized circumstances during the epicardial surgical procedure. Actually, in early trial of epicardial ablation, many testing techniques for complete conduction block have been applied but denied as their insensitivity when mapping epicardial. The situation doesn't change until Lockwood et al^23^ suggest separate narrow probe with small closely spaced bipolar electrodes rather than larger ones primarily designed for RF ablation. Then it becomes possible to analyze the potentials of sites on the opposite side of and close to the lesion line, and they also report the astonishing twenty‐one percent (3/14) of complete block after the first set of RF application. In other words, the reported survival rates of early studies have been underestimated, so as ours. And the early recurrence defined as recurrent AF/AFL/AT within 3 months of ablation is not rare in our study (16.13%), which also indicates the reconduction and residual gaps.

Then referring the ablation of ganglionated plexus which along with other mechanisms orchestrates the AF initiating and atrial autonomic remodeling, even though basic research underlines its central role during the procession of AF, there has always been limited evidence supporting this strategy.^24^ And it is reported that in animal model the ganglionated plexus activity could recover 4 weeks after the selective ablation.^25^ Moreover, the efficacy and safety of ganglionated plexus ablation for patients with persistent AF, enlarged left atrium or failed catheter ablation has been completely denied as the republished data of the AFACT study.^26^ And for now, ganglionated plexus ablation is no longer embraced by major lesion set, despite occasional mention.

Compared with other reported techniques for surgical epicardial ablation, besides the bipolar radiofrequency source, there are prominent advantages in our performed procedure. During the operation, the lesion site settled as the routs the navigators getting through and later confirmed when isolator dock in taking the linear motion guided by navigators. The ingenious set of symmetric lesion and subtle combination of Cardioblate Navigators (Medtronic) and Cardioblate Gemini‐s bipolar radiofrequency isolator (Medtronic) realize the isolation of PVs and most portion of posterior left atrial wall while the procedure is still quite reproducible. And meanwhile the procedural exclusion of jaw switching simplifies the operation and avoids the possible damage made around PVs. Actually, the benefits of alone linear motion come more, as it enables the application with smaller ports resulting in procedure less invasive and time‐consumptive. 113 min as mean operation time is far more promising than the given figures of other reported epicardial ablation techniques without mapping. And the more familiar performing surgeon gets with the procedure, the more time‐saving would it be. We compared the documented twenty‐nine cases splitting them into the earlier fifteen and the subsequent fourteen finding that the mean operation time declines from 135 ± 25 to 91 ± 24 min.

Hybrid ablation combines epicardial ablation, endocardial mapping, and catheter ablation first introduced by Pison et al^12^ was imposed as with higher efficiency for its advantage of selective linear lesion making, especially the one connected to mitral isthmus. However, in most studies, the long‐term performance of hybrid ablation fails the expectation sharing the long‐term success rate with epicardial ablation as around forty‐five percent when the follow‐up extends to 5 years.^27^, ^28^, ^29^, ^30^ Actually, no substantial improvements have been made with the tinsel inclusion of catheter‐based mapping and ablation when compared with epicardial ablation following Dallas lesion set and incorporating epicardial mapping. And there is the extra need for catheter induction doing more harm and prolonging the operation, as well as full hepatization that would increase the risk of bleeding leading to transfusion or even conversion to median sternotomy or re‐exploration, letting alone radiation, contrast medium, and extra financial cost.

The less invasiveness or higher effectiveness is a debating topic among physicians, as it is never easy to reach an equilibrium point. Even though early researchers have proven the limited value of right atrial lesions, a relatively new procedure including these lesions accepted as minimally invasive named as the endocardial Cox maze procedure is supported by many researchers.^7^, ^13^, ^31^, ^32^, ^33^ Despite the elaborate and ingenious design for lesion making, it is difficult to explain the differences between the minimally invasive Cox maze IV procedure and traditional surgical ablation after median sternotomy, as frequent suture and puncture, full heparinization, cardiopulmonary bypass, aortic cross‐clamp, and incision on the left atrium are all implicated during the procedure. And for now, the follow‐up of 1–2 years suggests one nose winning of minimally invasive CMIV procedure, while there are still limited articles addressing its long‐term efficiency, which could never reach 75% of the 5‐year success rate after one traditional Cox maze procedure with trauma of comparative scale.

In this study, the overall 5‐year arrhythmia‐free survival rate after one procedure is 45.43% in accord with Zheng et al.^28^ One evident shortcoming of this procedure is the absence of electrophysiological confirmation of bidirectional block of the lesions, which may partially lead to the instant postoperative recurrence. Without epicardial mapping, the operational success could be overestimated while the long‐term efficacy would be underestimated. And one study including periprocedural confirmation of ablation lesions obtains enhanced 1‐year efficacy of epicardial ablation with 86% one‐procedure success rate.^34^ Another possible shortcoming would be the incomplete isolation of the posterior wall of left atrium. As the symmetric lesion set circles parts of the posterior wall into the isolation area, there are remaining sources of reentry wavelets at large. And the linear lesion connecting the mitral isthmus is later incorporated. Thus, besides the initial 5‐year experience elucidated there are still jobs to do figuring out the long‐time efficacy of epicardial ablation with lesions also isolating the posterior wall of left atrium and perioperatively confirmed by mapping techniques. Anyway, for now, the long‐term result is way less than satisfactory so as other techniques, and there is no such consensus of standardized procedure for lone AF management. Therefore, it is essential for status quo informing the patients with all procedures in detail even the traditional invasive surgery, which should also include the causing trauma and long‐time efficacy.

There are limitations to this study. First, the limited number of enrolled patients in our center from 2011 to 2013 may increase the selection bias. Second, also due to the small sample size, further analysis of recurrence and complication events is impossible. Finally, ATa episodes could flee from monitoring of Holter tests let along daily ECG.

## 5 CONCLUSION

In summary, the applied mini‐invasive epicardial surgical procedure is acceptable in the long term as the 5‐year survival rate reaches 45.4%. And this shared procedure has the advantages of shorter operation time and less surgical trauma. Therefore, the epicardial ablation is quite recommendable for medical intervention of atrial fibrillation especially the lone one.

## CONFLICTS OF INTEREST

Dr. Yingqiang Guo and other authors have no financial or personal relationship with individuals or institutions that would inappropriately influence this work.

## AUTHOR CONTRIBUTIONS

All authors sufficiently contributed to the intellectual content, review of literature, and analysis of data. Each author has reviewed the final version of the manuscript and approves it for publication.

## ETHICAL APPROVAL

This study was approved by the Clinic and Biomedical Ethics Center of West China Clinic Medicine School Sichuan University, with informed consent obtained.

## CONSENT

Every patient enrolled to participation and publication.

## Data Availability

The datasets generated and analyzed during the current study are available from the corresponding author on reasonable request.
